# Sonic Hedgehog Mediates High Frequency-Dependent Deep Brain Stimulation for the Correction of Motor Deficits in a Parkinson’s Disease Model

**DOI:** 10.1007/s12264-024-01306-y

**Published:** 2024-11-06

**Authors:** Hui Zhang, Yujuan Su, Zhongwei Qu, Chunkui Zhang, Shaorong Ma, Xia Li, Yizheng Wang

**Affiliations:** 1 National Clinical Research Center for Aging and Medicine, Huashan Hospital, State Key Laboratory of Medical Neurobiology, Fudan University, Shanghai, 200040 China; 2 Laboratory of Neural Signal Transduction, Institute of Neuroscience, Chinese Academy of Sciences, Shanghai, 200031 China; 3 Center of Cognition and Brain Science, Beijing Institute of Basic Medical Sciences, Beijing, 100850 China

**Dear Editor,**

Deep brain stimulation targeting the subthalamic nucleus (STN-DBS) can relieve motor symptoms in patients with advanced Parkinson’s disease (PD). The efficacy of DBS is strongly dependent on stimulus frequency, typically exceeding 100 Hz [1]. However, the mechanism underlying this high-frequency dependence remains unelucidated. DBS likely acts *via* several, nonexclusive mechanisms, including local and network-wide electrical and neurochemical effects, modulation of oscillatory activity, synaptic plasticity, and potentially neuroprotection and neurogenesis. Nonetheless, none of these fully explains the high-frequency dependence and the rapid therapeutic action of DBS. Therefore, identifying the molecular mechanism underlying the high frequency-dependence of DBS can facilitate the development of next-generation PD treatments.

Sonic hedgehog (Shh) is a secreted protein. It binds to Patched (Ptch1) to relieve the Ptch1 inhibition of Smoothened (Smo), a transmembrane protein homologous to G-protein coupled receptors, thereby triggering downstream responses [2]. Shh is known as a mitogen or morphogen in the embryonic development of the central nervous system (CNS) [3]. In the mature CNS, Shh remains expressed by certain cell types, though its role has yet to be fully understood. Furthermore, dysregulation of Shh signaling in the brain leads to neurological disorders like autism spectrum disorder, depression, dementia, stroke, epilepsy, and PD [4–6]. In a marmoset model of PD exposed to 1-methyl-4-phenyl-1,2,3,6-tetrahydropyridine, Shh has been found to suppress midbrain dopaminergic neuron death and improve motor function [7]. Shh knockout from dopamine neurons results in PD-like locomotor deficits [8]. In PD patients, alterations in gene expression related to Shh signaling have been found [5]. Furthermore, the loss of primary cilia necessary for Shh signaling linked to leucine-rich repeat kinase 2 (LRRK2) mutations has been discovered in human postmortem PD brains [9]. Moreover, glutamate levels and glutamate transporter activity change following chronic 6-hydroxydopamine lesions [10]. Our previous study on adult mice revealed that Shh is quickly released to regulate extracellular glutamate levels by maintaining glutamate transporter membrane expression under pathological conditions, such as in epilepsy and ischemia [11, 12]. Furthermore, high-frequency stimulation, as opposed to low-frequency stimulation, induces the release of Shh in neurons [13]. Considering that Shh regulates glutamate levels and its high-frequency-dependent release, this study hypothesized that Shh mediates the high-frequency-dependent STN-DBS corrective effects on the PD model.

To this end, hemi-parkinsonian rodent models were established by injecting 6-hydroxydopamine hydrobromide into the unilateral striatum of mice (2 μg/μL, 2 μL) and the medial frontal bundle of rats (5 μg/μL, 3 μL). Motor deficits of the PD rodents were assessed with behavioral assays, including the apomorphine (APO)-induced contralateral rotation test and the balance beam test (Figs. S1A, B). Briefly, the APO-induced rotation test was conducted by counting full-body rotations in a transparent cylinder following APO injection. The balance beam test measured the time for rodents to traverse a beam. The loss of dopaminergic neurons in the substantia nigra compacta was evaluated by immunofluorescent staining for the anti-tyrosine hydroxylase antibody (Figs. S1C, D). In PD rodents, the electrode was implanted into the ipsilateral subthalamic nucleus (STN), which is one of the most effective DBS targets for PD patients. We found that STN-DBS at high frequency (100 Hz) significantly reduced the number of APO-induced contralateral rotations [14] and the time for passing the beam. In contrast, STN-DBS at low frequency (10 Hz) did not ameliorate these motor deficits (Fig. 1A, B for mice; S1E, S1F for rats). Subsequently, the role of Shh signaling in STN-DBS corrective effects in the PD rodents was explored using cyclopamine (Cyc) or Smoothened agonist (SAG), agents known to inhibit or stimulate Smoothened (Smo), respectively, a molecule known to be the key mediator of Shh signaling. Thirty minutes after intraperitoneal (i.p.) injection of Cyc, the corrective effects of DBS on APO-induced contralateral rotation and the time for passing the beam were blocked in the PD rodents (Fig. 1C, D for mice; S1G for rats). Cyc alone did not affect the PD motor deficits, as shown in Fig. S1H. Similar results were found following intracerebroventricular (i.c.v.) injection of Sant-1, another antagonist of Smo (Fig. S1I). Furthermore, activating Shh signaling by i.c.v. injection of SAG markedly reduced the number of rotations and the time for passing the beam to an extent similar to STN-DBS in the PD rodents (Fig. 1E, F for mice; S1J for rats). These results provided the initial evidence that Shh is likely released upon high-frequency STN-DBS and specifically mediates the corrective effects of DBS on motor deficits in PD rodents.

**Fig. 1:**
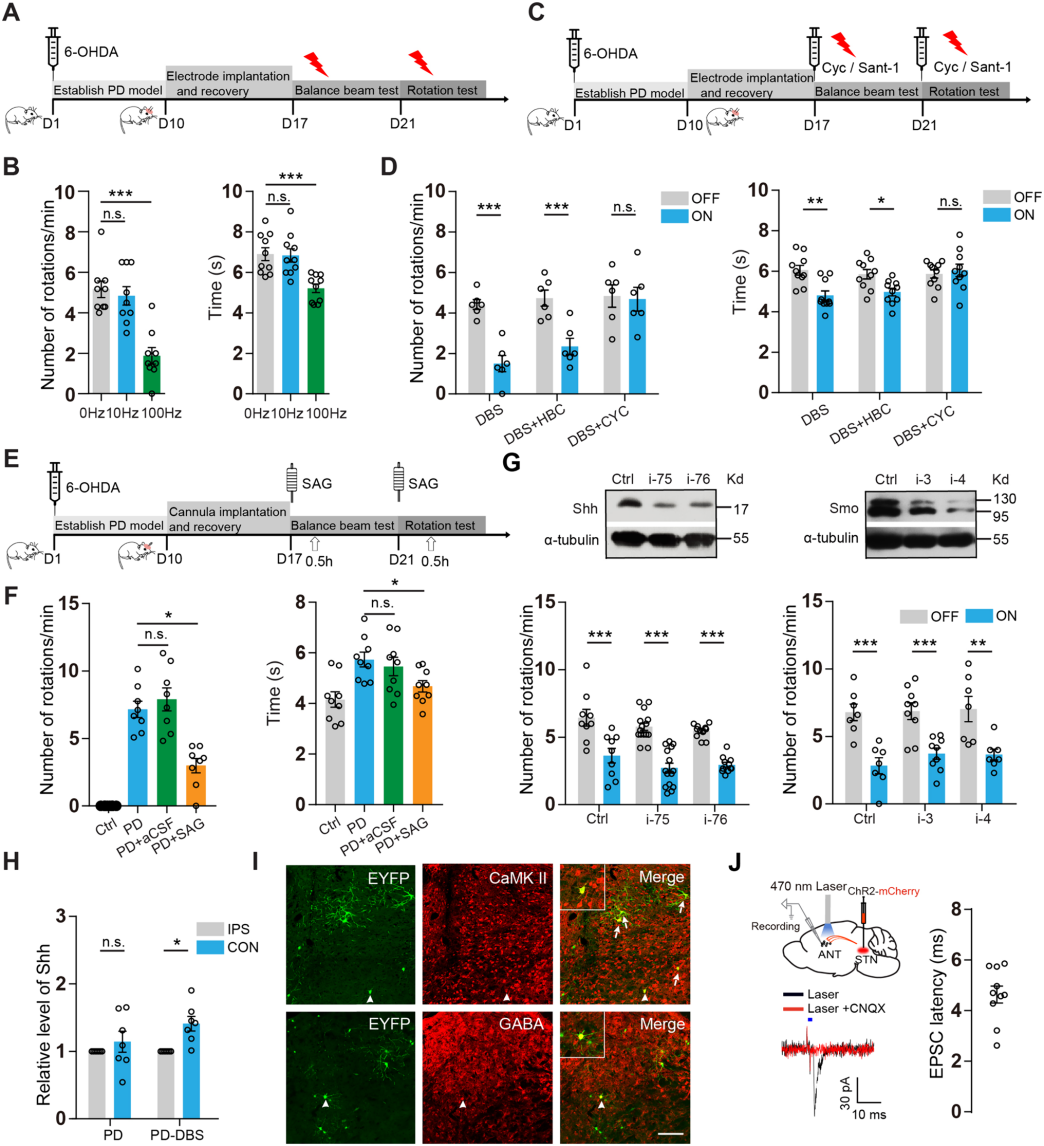
Shh release from the contralateral ANT modulates STN-DBS corrective effects on PD motor deficits. **A** Schedule of STN-DBS corrective effects on PD motor deficits. **B** The number of APO-induced rotations (left, *n =* 9 mice per group, one-way ANOVA with Dunnett’s *post-hoc* test) and the time for passing the beam (right, *n =* 10 mice per group, one-way ANOVA with Dunnett’s *post-hoc* test) after STN-DBS with indicated frequencies in PD mice. **C** Schedule of STN-DBS treatment effects on behaviors in PD rodents after injection of Cyc or Sant-1. **D** Effects of STN-DBS on the number of APO-induced rotations (left, *n =* 6 mice per group, two-way ANOVA with Bonferroni’s *post-hoc* test) and the time for passing the beam (right, *n =* 10 mice per group, two-way ANOVA with Bonferroni’s *post-hoc* test) after i.p. injection of Cyc (10 mg/kg) or the solvent (hydroxypropyl-beta-cyclodextrin, HBC) in the PD mice. **E** Schedule of the corrective effects of SAG on the PD motor deficits. **F** Number of APO-induced rotations (left, *n =* 8 mice per group, Kruskal-Wallis test with Dunn’s *post-hoc* test) and the time for passing the beam (right, *n =* 9 mice per group, one-way ANOVA with Bonferroni’s *post-hoc* test) after i.c.v. injection of SAG (5 μmol/L in 1 μL) or solvent (aCSF) in PD mice. **G** Representative immunoblots of Shh and Smo in cultured hippocampal neurons treated with LV-Shh-RNAi (upper, left) or LV-Smo-RNAi (upper, right). Effects of STN-DBS on the rotations after knocking down Shh in the STN the PD mice (lower, left, *n =* 9–15 mice per group, two-way ANOVA with Bonferroni’s *post-hoc* test) or Smo (lower, right, *n =* 7–9 mice per group, two-way ANOVA with Bonferroni’s *post-hoc* test). **H** Levels of Shh released from ipsilateral and contralateral ANT in hemi-parkinsonian mice with or without STN-DBS (*n =* 7 mice per group, Wilcoxon matched-pairs signed rank test). Shh levels in the contralateral ANT normalized to the ipsilateral levels in each mouse. **I** Representative images of EYFP-positive ANT cells stained with anti-CaMKII and anti-GABA antibodies. Inset: magnified views of arrowhead regions. Scale bars, 200 μm. *n =* 5 mice. **J** Schematic of whole-cell recordings of the functional connection between the STN and contralateral ANT (upper, left). Statistics of the evoked EPSC latency by optical stimulation in contralateral ANT slices (blue bars, 470 nm; 2 ms pulse width) (right). Representative traces of evoked EPSCs in ANT neurons with or without CNQX (10 μmol/L) (lower, left). *n =* 3 mice. For all behavioral experiments, all values are reported as the mean ± SEM from at least three independent experiments. Each circle represents a mouse, except in **J**, where each circle represents a cell. *P < 0.05, **P < 0.01, ***P < 0.001, n.s. no significant difference.

To further determine the role of Shh signaling in the corrective effects of STN-DBS on PD motor deficits, we investigated whether or how Smo stimulation in the ipsilateral STN participates in these corrective effects. The virus targeting Shh or Smo, whose knockdown efficiency was validated using Western Blot analysis in cultured hippocampal neurons, was injected into the STN (0.5 μL). Knocking down Shh or Smo in the ipsilateral STN did not impact the corrective effects of DBS on the rotational behavior (Figs. 1G, S2A-C). Since the STN projects to the internal globus pallidus (GPi) and anterior thalamic nucleus (ANT) [15], we examined the Shh levels in those nuclei *via* the enzyme-linked immunosorbent assay. The results revealed that Shh levels were increased only in the contralateral ANT after ipsilateral STN-DBS in hemi-parkinsonian mice (Figs. 1H; S2D). Consistent with our findings, Shh expression in the ANT was previously detected by *in situ* RNA hybridization as shown in the Allen Mouse Brain Atlases (https://mouse.brain-map.org/gene/show/20186). Then, we asked whether the STN also projects to the contralateral ANT. Anterograde tracing adeno-associated virus (AAV) expressing enhanced yellow fluorescent protein (EYFP) (rAAV-CaMKII-EYFP, 0.1 μL) was injected into the unilateral STN. EYFP-positive fibers were observed in the ipsilateral external globus pallidus (GPe), GPi, and ANT, and were also found in the contralateral ANT (Fig. S2E). Furthermore, AAV-mediated anterograde trans-synaptic tracing by injecting AAV-Cre (0.1 μL) and a Cre-dependent AAV encoding EYFP (0.1 μL) into the STN and the contralateral ANT was applied to verify the structural connection between the STN and contralateral ANT. EYFP-positive cells were found in the contralateral ANT. Further analysis revealed that ANT cells were mainly co-labeled with NeuN, a neuronal marker. In addition, the EYFP-positive neurons were co-labeled mainly with Ca^2+^/calmodulin dependent protein kinase II (CaMK II) (74.27% ± 3.66%), but less with GABA (23.6% ± 2.57%) (Figs. 1I; S2F). ChR2-assisted whole-cell electrophysiological recordings confirmed the functional connection between the STN and contralateral ANT. Three weeks after injecting rAAV-CaMKII-hChR2(H134R)-mCherry (0.1 μL) into the ipsilateral STN, optical stimulation among mCherry-positive fiber terminals in contralateral ANT slices evoked excitatory postsynaptic currents (EPSCs). The average latency of the evoked EPSCs in contralateral ANT neurons was 4.635 ± 0.338 ms. Moreover, the evoked EPSC was blocked when CNQX (10 μmol/L), a competitive AMPA-type glutamate receptor antagonist, was added to the artificial cerebrospinal fluid (aCSF) (Fig. 1J). Altogether, these results suggested that STN projects to the bilateral ANT and ipsilateral STN-DBS cause Shh release in the contralateral ANT.

The next experiment tested whether the contralateral ANT is responsible for the corrective effects of ipsilateral STN-DBS on motor deficits in PD mice (Fig. S3A). Down-regulating Shh (Fig. 2A, B) or Smo (Figs. S3B, C) in the contralateral ANT both blocked the corrective effects of STN-DBS on the rotational behavior and the time for passing the beam. Considering that knocking out Shh affects the early development of the CNS, and the lack of an ANT-specific promoter, we inhibited Shh signaling using a neutralizing antibody (5E1) [16] in the contralateral ANT to further explore the role of Shh release and Smo stimulation in the corrective effects of DBS. As shown in Fig. 2C, the corrective effects of DBS on PD were blocked by 5E1 but not by its heat-inactivated form (HI-5E1). Moreover, injecting Shh, but not its heat-inactivated form (HI-Shh), into the contralateral ANT greatly reduced the number of rotations in the PD mice (Fig. 2D). Furthermore, injecting SAG to activate Shh signaling in the contralateral ANT corrected the motor deficits in the PD mice (Fig. 2E). Taken together, these results suggested that Shh signaling in the contralateral ANT indeed mediates the correction of PD motor deficits by STN-DBS.

**Fig. 2:**
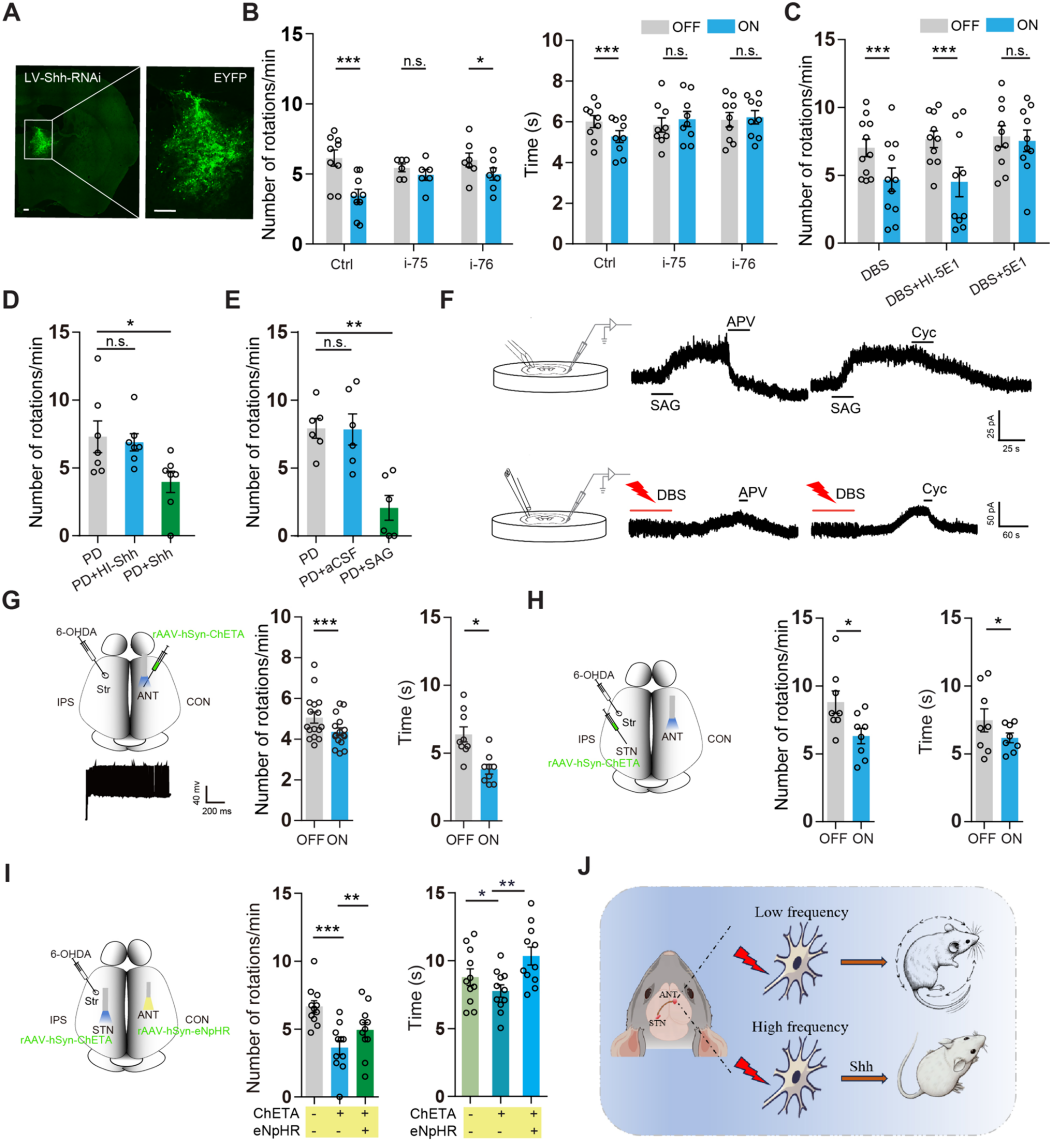
Contralateral ANT activity is necessary for STN-DBS correction of PD motor deficits. **A** Representative image of LV-Shh-RNAi expression in the contralateral ANT. Scale bars, 200 μm. **B** STN-DBS effects on the number of rotations (left, *n =* 6–9 mice per group, two-way ANOVA with Bonferroni’s *post-hoc* test) and the time for passing the beam (right, *n =* 9 mice per group, two-way ANOVA with Bonferroni’s *post-hoc* test) after knocking down Shh in the contralateral ANT of PD mice. **C** STN-DBS effects on the rotations 30 mins after heat-inactive 5E1 (HI-5E1) or 5E1 (0.1 μg/μL) injection into the contralateral ANT of PD mice (*n =* 10–11 mice per group, two-way ANOVA with Bonferroni’s *post-hoc* test). **D** The number of rotations 30 mins after injecting heat-inactivated Shh (HI-Shh) or Shh (0.1 μg/μL) into the contralateral ANT of the PD mice (*n =* 7 mice per group, one-way ANOVA with Dunnett’s post-hoc test). **E** The number of rotations 30 mins after injecting SAG (5 μM) into the contralateral ANT in PD mice (*n =* 6 mice per group, one-way ANOVA with Dunnett’s *post-hoc* test). **F** Schematic of the homemade flow-pipe perfusion apparatus, which was positioned at ~30° and 1 mm from the recording pipette, and representative current traces elicited by SAG (50 μmol/L), APV (50 μmol/L), or Cyc (100 μmol/L) in ANT slices (upper). Schematic of homemade flow-pipe perfusion, electrical stimulation apparatus, and representative current traces elicited by high-frequency electrical stimulation, APV (50 μmol/L) or Cyc (100 μmol/L) in ANT slices (lower). *n =* 3 mice. **G** Schematic of virus injection and optical fiber implantation in PD mice (upper, left) and a representative trace of spikes after activating EYFP-positive ANT neurons by 470 nm optical stimulation at 100 Hz (lower, left). The number of rotations (middle, *n =*16 mice per group, paired two-sided *t*-test) and the time for passing the beam (right, *n =* 9 mice per group, paired two-sided *t*-test) in PD mice after optical stimulation at 100 Hz in the contralateral ANT. **H** Schematic of virus injection and optical fiber implantation into the ipsilateral STN and contralateral ANT of PD mice (left). The number of rotations (middle, *n =* 8 mice per group, paired two-sided *t*-test) and the time for passing the beam (right, *n =* 8 mice per group, paired two-sided *t*-test) after high-frequency optical stimulation (470 nm, 100 Hz). **I** Schematic of virus injection and optical fiber implantation into the ipsilateral STN and contralateral ANT of PD mice (left). The number of rotations (middle, *n =* 11 mice per group, one-way ANOVA with Bonferroni’s *post-hoc* test) and the time for passing the beam (right, *n =* 11 mice per group, one-way ANOVA with Bonferroni’s *post-hoc* test) after high frequency (100 Hz) 470 nm optical stimulation in the ipsilateral STN followed by 590 nm optical stimulation in the contralateral ANT. **J** A working model of the role of Shh in the high-frequency-dependent STN-DBS correction on the motor deficits in the PD model. For all behavioral experiments, all values are reported as the mean ± SEM from at least three independent experiments. Each circle represents a mouse. *P <0.05, **P < 0.01, ***P < 0.001, and no significant difference (n.s.).

Our previous work showed that Shh rapidly regulates extracellular glutamate levels in hippocampal neurons [11], and glutamate levels are reflected by NMDAR-mediated currents in whole-cell recordings. Subsequent electrophysiological experiments revealed that the application of SAG or DBS to ANT slices does evoke a current, which is blocked by Cyc or APV, a selective NMDA receptor antagonist (Fig. 2F). These results support the hypothesis that the corrective effects of DBS likely result from the rapid regulation of extracellular glutamate levels by Shh, affecting neuronal activity. To test this assumption, we optically stimulated the contralateral ANT neurons expressing rAAV-hsyn-ChETA-EYFP (0.1 μL) and found that the evoked action potentials were able to follow 100 Hz optical stimulation (Fig. 2G, left). In addition, the behavioral tests revealed that optical stimulation at 100 Hz in the contralateral ANT reduces the number of APO-induced rotations and the time for passing the beam in the PD mice (Fig. 2G, middle and right). To further investigate the role of the STN-contralateral ANT circuit in the PD motor deficits, virus expressing ChETA (0.1 μL) was injected into the STN, and a fiber was implanted in the contralateral ANT. The number of APO-induced rotations and the time for passing the beam were reduced after high-frequency optical stimulation (Fig. 2H), compared to low-frequency (Fig. S3D). Furthermore, the corrective effects of high-frequency optical activation on the STN were blocked when optical stimulation was applied to the contralateral ANT expressing eNpHR (rAAV-hSyn-eNpHR-YFP, 0.1 μL) (Fig. 2I). Low-frequency stimulation did not affect the behaviors, irrespective of eNpHR expression in the ANT neurons (Fig. S3E).

Furthermore, we investigated the role of STN neurons projecting to the contralateral ANT in the corrective effects of STN-DBS on PD mice. Expressing eNpHR (0.1 μL) in the ipsilateral STN and optically stimulating the fibers in the contralateral ANT partly blocked the STN-DBS effects (Fig. S3F); in contrast, optically stimulating the fibers in the ipsilateral ANT did not (Fig. S3G). These results suggested that neuronal fibers projecting from the STN to the contralateral ANT can medicate the STN-DBS effects on the behaviors of PD mice. Collectively, these results support the conclusion that increased neuronal activity in the STN-contralateral ANT circuit plays an important role in the STN-DBS correction of PD motor deficits.

The key finding of our study was that under high frequency (100 Hz) rather than low frequency (10 Hz) conditions, Shh is released in the contralateral ANT to mediate the STN-DBS corrective effects on motor deficits in hemi-parkinsonian rodents. Several lines of experimental results support this conclusion. First, ipsilateral STN neurons project to bilateral ANT neurons. Second, the contralateral ANT neurons release Shh in a high frequency-dependent manner and mediate the corrective effects of ipsilateral STN-DBS. Third, activating the contralateral ANT, especially the STN-ANT circuit, by high-frequency optical stimulation corrects the motor deficits in PD mice. Finally, inhibiting the activation of fibers projecting from the STN to the contralateral ANT partly blocks the effects of STN-DBS on motor deficits in PD mice. The current results led us to propose a model in which Shh is released in the contralateral ANT after high-frequency STN-DBS, mediating its corrective effects on motor changes in hemi-parkinsonian rodents (Fig. 2J).

An important finding of the current study is that Shh release in adult mice is high-frequency dependent. Previous work from our laboratory has shown that high-frequency stimulation induces the release of Shh from hippocampal neurons by increasing intracellular Ca^2+^ levels, triggering the exocytosis of synaptic vesicles [13]. Moreover, in conditions with intense neuronal activity, such as epilepsy and acute cerebral ischemia, Shh release is evident. It is thus possible that STN-DBS induces changes in Ca^2+^ and neuronal activity which are crucial for Shh release. It has been reported [17] that activating Smo in striatal cholinergic interneurons (CINs) reduces L-Dopa-induced dyskinesia, supporting the role of Shh motor control circuits. However, it remains unclear whether ANT neurons project to CINs and activate Smo *via* Shh. Future studies are needed to explore this possibility to understand the effects of the ANT on motor control.

The mechanisms underlying DBS are complex, involving regulation at different levels. For example, at the molecular level, DBS affects neurotransmitter release or regulates neuronal excitability through voltage-dependent ion channels. Our results from optogenetics and *ex vivo* electrophysiological recordings suggest that Shh influences neuronal activity through glutamate, contributing to improvements in the motor deficits of PD models. It has been reported that Shh signaling affects the ability of astrocytes to clear glutamate [18], hence modulating excitatory neurotransmission. Thus, it is possible that Shh mediates the therapeutic effects of DBS by modulating glutamate levels through astrocytes. The current results suggest a potential integration of glutamate, astrocytes, and Shh, offering a more comprehensive understanding of how DBS alleviates motor symptoms in PD patients. In addition, Shh is involved in synaptic plasticity [19], which may contribute to the long-term benefits of DBS. Further research is required to dissect these pathways and clarify the specific contributions of each to the therapeutic outcomes reported with STN-DBS.

Several limitations need to be noted regarding the present study. First, given the complexity of DBS mechanisms, it is important to consider alternative pathways that contribute to the observed effects. Besides the ANT, the STN projects to multiple basal ganglia regions involved in motor control (GPe, GPi, and substantia nigra), which could confound results by affecting multiple brain regions rather than just the STN-ANT pathway. For instance, the modulation of glutamatergic and GABAergic transmission within these other regions could interact with the Shh signaling pathway, leading to a more complex network of influences that contribute to the therapeutic outcome. To address these potential confounding factors, future studies should aim to apply more targeted approaches such as optogenetics or chemogenetics to selectively manipulate specific pathways. Second, while the ANT mediates the STN-DBS correction of motor deficits in PD models, the precise role of the ANT in motor symptoms, such as bradykinesia, tremors, and gait impairments, remains unclear. Future studies are needed to investigate STN-DBS effects in animal models such as non-human primates, which share closer anatomical and functional similarities with humans, to provide more comprehensive data and bridge the gap between preclinical research and clinical application. Third, due to the technical limitations of virus injection, possible projections from nuclei surrounding STN to the bilateral ANTs and nearby nuclei contributing to the observed effects cannot be ruled out. Finally, the mechanism of Shh release in contralateral ANT after ipsilateral STN-DBS warrants further exploration.

Taken together, our work highlights the crucial role of Shh signaling in mediating the therapeutic effects of STN-DBS on motor deficits in hemi-parkinsonian rodents. This positions Shh as a potential molecular target for developing non-invasive treatments, such as ‘chemical DBS’, that mimic high-frequency DBS. Drugs like SAG, which activates Shh signaling, could replicate the benefits of STN-DBS without surgery, offering a less invasive option for PD patients and eliminating surgical risks. Future research is needed to validate these findings in more complex models to explore the efficacy and safety of chemical DBS agents.

## Supplementary Information

Below is the link to the electronic supplementary material.

Supplementary file1 (PDF 902 KB)
