# Supplementary material for: Sonic Hedgehog Mediates High Frequency-Dependent Deep Brain Stimulation for the Correction of Motor Deficits in a Parkinson’s Disease Model
Source: Neurosci Bull. 2024 Nov 6;40(11):1732–8. doi: 10.1007/s12264-024-01306-y (PMC11607294; doi:10.1007/s12264-024-01306-y)
Supplement: Supplementary file 1 — Supplementary file1 (PDF 902 KB) [file 12264_2024_1306_MOESM1_ESM.pdf]

Supplementary Materials

Materials and Methods

Animals

Male C57/BL6 mice aged 5–8 weeks and male Sprague-Dawley (SD) rats weighing 250–350 g or at embryonic day 18 (E18) were housed in groups of three to five (mice) or two (rats) per cage, with a 12-h light/dark cycle, constant temperature (20 ± 2°C), and humidity (40%–60%). They had *ad libitum* access to food and water. All animal handling, surgery, and behavioral tests were carried out in accordance with the Animal Care and Use Committee of Huashan Hospital of Fudan University, ensuring that all procedures adhered to strict ethical guidelines. Briefly, we followed the principles of the 3Rs (Replacement, Reduction, Refinement) to minimize animal use and suffering. For Replacement, given the nature of our research on PD and DBS, animal models were essential to study the complex interactions and effects within a living organism. For Reduction, we used the smallest number of animals necessary to obtain data, including careful planning and design of experiments to ensure efficiency and avoid unnecessary use of animals. For Refinement, we refined our experimental procedures to minimize pain and distress. All surgical procedures were performed under anesthesia.

Primary Cell Culture

Primary hippocampal neurons were cultured following a method described previously [1]. Briefly, hippocampal neurons were isolated from E18 SD rat brains and seeded into six-well plates coated with 50 µg/mL poly-D-lysine at a density of 7×10^4 cells/cm^2. The cells were incubated in a humidified atmosphere with 5% CO_2 at 37°C. On the third day, 5-Fluoro-2'-deoxyuridine (7.5 µg/mL) and uridine (17.5 µg/mL) were added to the plates to suppress the growth of glial cells. The virus was added on the fifth day to knock down gene expression. Cells were cultured in the neurobasal medium (Gibco, 21103049) supplemented with 2% B-27 (Gibco, 17504044) and 2 mmol/L glutamine (Gibco, 35050061) for 10 days before use. During the 10-day culture period, the medium was partially changed every 3–4 days to maintain optimal growth conditions. The effectiveness of the viral knockdown was then evaluated using Western blotting.

Rodent Models of PD

The protocol used to establish hemi-parkinsonian rodent models with 6-OHDA was similar to the previous report[2]. Briefly, desipramine (Sigma, D3900) was intraperitoneally (i.p.) injected at a dose of 25 mg/kg for mice and 40 mg/kg for rats to increase the selectivity of 6-OHDA-induced lesions. The rodents were anesthetized with 0.7% pentobarbital sodium (w/v) (70 mg/kg), and then 6-OHDA (2 μg/μL and 2 μL for mice in the striatum, 5 μg/μL and 3 μL for rats in the MFB) dissolved in 0.9 % sterile saline containing 0.2% ascorbic acid (Sigma, H4381) was injected at the stereotaxic coordinates: (in mm) striatum, AP: 0.4, ML: 1.8, DV: −3.5); MFB (AP: −4.3, ML: 1.5, DV: −7.6) at a rate of 0.1 μL/min. The syringe was left in place for 5 min to allow the drug solution to diffuse into the nucleus. Apomorphine (0.5 mg/kg) was i.p. injected to induce contralateral rotation after three weeks. Only animals exhibiting >40 rotations in 10 min were selected for further experimental studies. After conducting all experiments, we evaluated the loss of dopaminergic neurons in the substantia nigra compacta (SNc) using immunostaining for anti-tyrosine hydroxylase (TH) antibody.

Surgery

After anesthesia, PD rodents were secured on a stereotaxic frame (RWD Instruments, 68044), and the skull was adjusted in parallel to the reference panel. A single-guide cannula (RWD Instruments, 62001) was implanted 300 μm above the center of either the ANT or lateral ventricle based on stereotaxic coordinates (ANT (mice): AP: −0.7, ML: 0.8, DV: −3.2, (rats) AP: −1.65, ML: 1.25–1.6, DV: −5.0; Lateral ventricle (mice): AP: −0.3, ML: 1.0, DV: −2.2, (rats) AP: −0.8, ML: 1.5, DV: −4.8). The skull surface was then covered with dental cement, secured with three anchoring screws (1 mm in diameter) to hold the cannula in place. The animals were allowed to recover from surgery for at least 7 days before conducting behavioral tests to ensure full recovery and minimize stress. For drug administration, drugs or their solvents were pumped into the targeted nuclei at a rate of 0.1 μL/min using syringe pump system (RWD Instruments, R404) for 30 min to 1 h before behavioral testing. Only data from animals with correctly placed implants were included in the analysis.

For DBS, homemade electrodes (200 μm in diameter) were used to deliver electrical stimulation to the STN (AP: −1.9, ML: 1.5, DV: −4.5) *via* two parallel tungsten wires. The implantation procedure was similar to that described above, and STN-DBS was applied after a one-week recovery period. The current intensity was controlled by a constant-current isolated stimulator, and stimulation

protocols were synchronized by Master-8 (A.M.P.I., Israel). High-frequency stimulation at 100 Hz is referred to as DBS unless stated otherwise. The pulse width was set at 100 μs, and the current intensity ranged from 100–500 μA, determined by the maximum value that did not result in abnormal dyskinetic movements.

Virus Preparation

5-8-week-old mice were used for virus injection. Briefly, each mouse was anesthetized with 0.7% pentobarbital sodium (70 mg/kg) and positioned in a stereotaxic frame. The skull was exposed by a small incision, and then a small hole was drilled at the target coordinates using a dental drill. Viruses were injected into either the STN or ANT using a Hamilton syringe coupled with a pulled glass pipette. The injection was performed at a rate of 0.02 μL/min, with a volume of 100 nL per injection site, unless otherwise specified. The stereotaxic coordinates for the STN were AP: −1.9, ML: 1.5, DV: −4.6, and for the ANT, AP: −0.7, ML: 0.8, DV: −3.5. After the injection, the pipette was left in place for 10 min to allow proper diffusion of the virus into the targeted region. Following this period, the pipette was carefully removed to minimize potential backflow of the virus. The incision was then closed with surgical sutures, and the mice were allowed to recover from anesthesia on a heating pad to maintain body temperature. After three weeks, behavioral experiments, electrophysiological recordings, or immunostaining were performed. Only animals with correct injection placement were used for data analysis.

The viruses were as follows:

rAAV2/9-CaMKIIα-EYFP (STN, titre:1.19 × 10^{13} viral genomes (vg)/mL, 0.1 μL; BrainVTA PT-0102), rAAV2/1-CaMKIIα-Cre (STN, titer: 1.14 × 10^{13} vg/mL, 0.1 μL, BrainVTA PT-0220), rAAV-Ef1α-Dio-EYFP (ANT, titer: 5.35× 10^{12} vg/mL, 0.1 μL, BrainVTA PT-0012), rAAV-CaMKIIα-hChR2(H134R)-mCherry (STN, titre:2×10^{12} vg/mL, 0.1 μL, BrainVTA PT-0279), rAAV-CaMKIIα-ChETA-EYFP (STN, titre:3.2×10^{12} vg/mL, 0.1 μL, BrainVTA PT3110), rAAV-hSyn-eNpHR-YFP (ANT, titer: 3.21×10^{13} vg/mL, 0.1 μL BrainVTA PT-0724). The lentivirus (LV)-based RNA interference against Shh (i-75 and i-76) and against Smo (i-3 and i-4) (>10^{8} U/mL) was packaged (GeneChem, China) and the sequences were: shShh-i-75: GGGTCTACTATGAATCCAAAG, shShh-i-76: GGTGCAAAGACAAGTTAAATG, shSmo-i-3: AGGCAGAGATCTCCCCAGAGTT,

shSmo-4: AGCAGATGG CACCATGAGAATTT. Two different viruses were injected into the STN and ANT, each with a volume of 500 nL.

Optogenetic Manipulation

To optogenetically manipulate neuronal activity *in vivo*, an optical fiber (200 μm in diameter) was placed 300 μm above the center of the STN or ANT in hemi-parkinsonian mice expressing AAV-based ChETA or eNpHR. Three weeks after virus injection, behavioral experiments were performed. In mice expressing ChETA or EYFP, a 470 nm blue laser was used to activate the neurons at either 100 Hz or 10 Hz during behavioral tests. In mice expressing eNpHR or EYFP, a 590 nm yellow laser was used to inhibit the neurons during behavioral tests. Laser output was controlled at an intensity of 5 mW/mm^2.

Immunofluorescence Staining

After anesthesia, mice were transcardially perfused with 0.9% sterile saline followed by 4% paraformaldehyde (PFA). Each brain was extracted and post-fixed in 4% PFA for 6 h at 4°C. Following post-fixation, each brain was transferred into 20% sucrose until it sank, and then into 30% sucrose for 36–48 h for cryoprotection. Coronal sections were cut at 40 μm on a freezing microtome (Leica, CM1950) for immunostaining. The sections were pre-incubated in blocking buffer containing 3% bovine serum albumin and 0.3% Triton X-100 for 2 h at room temperature (RT), followed by overnight incubation at 4°C with the relevant primary antibodies. The sections were rinsed in phosphate-buffered saline (PBS; 3 × 10 min) and then incubated with fluorescent secondary antibodies for 2 h at RT. The sections were rinsed again in PBS (3 × 5 min), followed by mounting with an aqueous mounting medium (Sigma, F4680). Immunofluorescence images were captured on a laser-scanning confocal microscope (Nikon A1R, Japan). The primary and secondary antibodies were both diluted with PBS containing 3% bovine serum albumin and 0.3% Triton X-100. The primary antibodies used for staining were as follows: rabbit-anti-TH: (Millipore, AB152, 1:1000), mouse-anti-NeuN (Millipore, MAB377, 1:800), rabbit-anti-CaMKII (Abcam, ab34703, 1:1000), rabbit-anti-GABA (Sigma, A2052, 1:1000), and mouse-anti-Cre (Millipore, MAB3120, 1:1000). The secondary antibodies used for staining were as follows: Alexa fluor 488- or 633- conjugated goat anti-rabbit or

anti-mouse (A11008, A11001, A21050), Alexa fluor 546- conjugated donkey anti-rabbit (A10040), from Invitrogen, all at 1:2000.

Western Blot

To extract total proteins from cultured hippocampal cells, an 8% sodium dodecyl sulfate (SDS) lysis buffer (2% SDS, 10% glycerol, 0.1 mmol/L dithiothreitol, and 0.2 mol/L Tris-HCl, pH 6.8) was used. Protein aliquots from each sample were separated by 8% SDS-polyacrylamide gel electrophoresis and transferred onto a polyvinylidene fluoride membrane. The membrane was then blocked with 3% non-fat milk in PBS at RT for 2 h and subsequently incubated overnight at 4°C with the primary antibodies (rabbit-anti-Shh: Cell Signaling 2287,1:1000, rabbit-anti-Smo: Abcam, ab72130, 1:1000, and mouse-anti-α-tubulin: Santa Cruz, sc47778, 1:1000). After rinsing in Tris-buffered saline Tween (3 × 10 min), the membrane was incubated with horseradish peroxidase (HRP)-conjugated secondary antibodies at RT for 2 h. The protein bands were visualized using the ECL system (Tanon Inc., 5200) with Luminata Crescendo Western HRP. The grayscale of each protein band was quantified using ImageJ software, allowing for precise measurement of protein expression levels.

ELISA

To determine Shh levels in the GPi and ANT, nuclei were extracted from PD mice immediately after STN-DBS or control. The tissue was incubated in 450 μL of artificial cerebrospinal fluid (aCSF) containing heparin for 20 min. The tissue was gently macerated with a manual stir bar and then centrifuged at 4°C for 10 min. The Shh level was determined by the corresponding ELISA kit (R&D, MSHH00). Briefly, 400 μL of supernatant was applied to wells coated with anti-Shh antibodies and incubated for 2 h, followed by the addition of Shh-N Conjugate, which was also incubated for 2 h. The substrate solution was incubated for 30 min, followed by a stop solution. Washing the well 5 times during each step was necessary for good performance, and all procedures were performed at RT. A microplate reader (BioTek, USA) was set to 450 nm for measuring absorbance and 540 nm for wavelength correction. ELISA Calc software was used to calculate standard curves by generating a four-parameter logistic curve fit. The standard curve was linear within the range used (0–500 pg/mL), and the Shh level in experimental samples was always within the linear range of the standard curve.

Behavioral Assays

Apomorphine-induced Rotation Test The apomorphine-induced rotation test was applied following the method described previously [3]. Briefly, mice were injected with apomorphine (0.5 mg/kg i.p.) and placed in a transparent cylinder 60 cm in diameter. The number of full-body rotations was counted 3 min after injection for a duration of 10 min. Only complete 360° turns were included in the count. The average number per minute was calculated to assess the motor deficits in PD rodents.

Balance Beam Test The balance beam test was conducted following the method described previously [4, 5] with a modification. Briefly, the apparatus consisted of a 1-meter-long round beam12 mm in diameter, placed 50 cm above the ground. At one end of the beam, a black box containing nesting material was placed as a destination, while a lamp at the origin side served as an aversive stimulus. Prior to testing, mice were trained for 3 days to acclimate to the apparatus. During training, each mouse was allowed to traverse the beam three times daily with 10-min intervals between trials. The time taken to pass over the beam was recorded as the measure of the mobility of PD rodents.

Whole-cell Electrophysiological Recordings

Slice Preparation

Whole-cell electrophysiological recordings were conducted following the method described previously [6]. Briefly, the mouse was anesthetized and decapitated, and the brain was dissected as quickly as possible. Coronal slices containing the ANT (300 μm thick) were cut on a vibratome in oxygenated ice-cold sucrose-based dissection solution ((in mmol/L): 213 sucrose, 2.5 KCl, 2 $MgSO_4$, 2 $CaCl_2$, 1.25 NaH_2PO_4, 26 $NaHCO_3$, and 10 glucose), followed by incubation, recovery in a recovery chamber at 34°C for 40 min, and then transfer to RT in the oxygenated aCSF ((in mmol/L):126 NaCl, 2.5 KCl, 2 $MgSO_4$, 2 $CaCl_2$, 1.25 NaH_2PO_4, 26 $NaHCO_3$ and 25 glucose, pH 7.2, 290-310 mOsm). All recordings were performed at RT and slices were superfused at 1.5-2 mL min^{-1} with aCSF, which was continuously bubbled with 95% O_2 + 5% CO_2 during experiments.

In Vitro Electrophysiological Recording

To determine the functional connection between the STN and the contralateral ANT, a mouse injected with AAV-based ChR2 (H134R) into the unilateral STN was prepared. Evoked EPSCs in ANT neurons were recorded in voltage-clamp mode at a holding potential of -70 mV with blue light (470 nm, 0.1 Hz, 2 ms). CNQX (Abcam, 120044, 10 μmol/L) was added to the aCSF to confirm the excitatory connection. To verify the function of AAV-based ChETA, whole-cell current clamp recordings were conducted to examine spikes in the ANT slice with optical stimulation (470 nm, 100 Hz) of EYFP-positive neurons. An intracellular solution containing (in mmol/L): 140 potassium gluconate, 3 KCl, 2 $MgCl_2$, 10 HEPES, 0.2 EGTA, and 2 Na_2ATP was used in the above two trials. Extracellular glutamate was detected whole-cell recordings following the method described previously [7]. Briefly, the voltage-clamp mode at a holding potential of +40 mV was applied to record NMDAR-mediated currents in the ANT slice. Picrotoxin (Abcam, ab120315, 100 μmol/L), NBQX (MedChemExpress, HY-15068, 10 μmol/L), TTX (0.5 μmol/L), and D-serine (Solarbio, S7120, 10 μmol/L) were added to the aCSF and the internal solution consisted of the following (in mM): 135 Cs-methanesulfonate, 8 NaCl, 10 HEPES, 10 Cs-BAPTA, 4 Mg-ATP, and 0.4 Na-GTP. SAG (MedChemExpress, HY-12848, 50 μmol/L, 1 μL), APV (Abcam, ab120003, 50 μmol/L, 1 μL), and Cyc (Selleckchem, S1146, 100 μmol/L, 1 μL) were applied using a home-made perfusion apparatus positioned above the ANT slice. Electrical stimulation was delivered by a tungsten bipolar stimulating electrode (FHC, Bowdoin, ME).

For all recordings, borosilicate glass pipettes were fabricated on a micropipette puller (Sutter Instruments) with a resistance of 3.5–5 MΩ. Neuronal leak currents for analysis were <100 pA. Data were acquired using HEKA EPC10 (HEKA, Germany), sampled at 10 kHz, and filtered at 2 kHz. Off-line analysis was done using Patchmaster (HEKA, Germany). The cell series resistance was <30 MΩ. The data that showed >20% change in series resistance were excluded from the analysis.

Statistical Analysis

GraphPad Prism was used for analyses, and data are presented as the mean ± SEM. Paired two-tailed *t*-tests were used to compare the means of two groups that followed a Gaussian distribution and had a similar amount of variance. Welch's *t*-test was used when two groups followed a normal distribution but did not have the same variance. The Mann-Whitney U test (unpaired data) and Wilcoxon matched-

pairs signed rank test (paired data) were applied only when the two groups were not normally distributed. When comparing the means of >3 independent groups, we used one-way analysis of variance (ANOVA) or two-way ANOVA containing one or two factors, respectively, for Gaussian distribution data with a similar amount of variance. Dunnett's *post-hoc* test was used for multiple comparisons between treatment and control groups; otherwise, we used Bonferroni's *post-hoc* analysis for multiple group comparisons. In contrast, we used the Kruskal-Wallis test with Dunn's *post-hoc* test to compare the medians of >3 independent groups when the data were not normally distributed. In cases where data could not be collected due to animal mortality or other unforeseen circumstances, we adjusted our data analysis methods to account for these missing values. *P <0.05, **P <0.01, ***P <0.001, n.s. no significant difference.

References

1. Brewer GJ, Torricelli JR, Evege EK, Price PJ. Optimized survival of hippocampal neurons in B27-supplemented Neurobasal, a new serum-free medium combination. J Neurosci Res 1993, 35: 567–576.
2. Tieu K. A guide to neurotoxic animal models of Parkinson's disease. Cold Spring Harb Perspect Med 2011, 1: a009316. doi:10.1101/cshperspect.a009316.
3. Kelly PH. Unilateral 6-hydroxydopamine lesions of nigrostriatal or mesolimbic dopamine-containing terminals and the drug-induced rotation of rats. Brain Res 1975, 100: 163–169.
4. Allbutt HN, Henderson JM. Use of the narrow beam test in the rat, 6-hydroxydopamine model of Parkinson's disease. J Neurosci Methods 2007, 159: 195–202.
5. Luong TN, Carlisle HJ, Southwell A, Patterson PH. Assessment of motor balance and coordination in mice using the balance beam. J Vis Exp 2011,49. doi: 10.3791/2376.
6. Zhang H, Zhang C, Qu Z, Li B, Su Y, Li X, *et al.* STN-ANT plasticity is crucial for the motor control in Parkinson's disease model. Signal Transduct Target Ther 2021, 6: 215.
7. Herman MA, Jahr CE. Extracellular glutamate concentration in hippocampal slice. J Neurosci 2007, 27: 9736–9741.

Supplementary Figure Legends

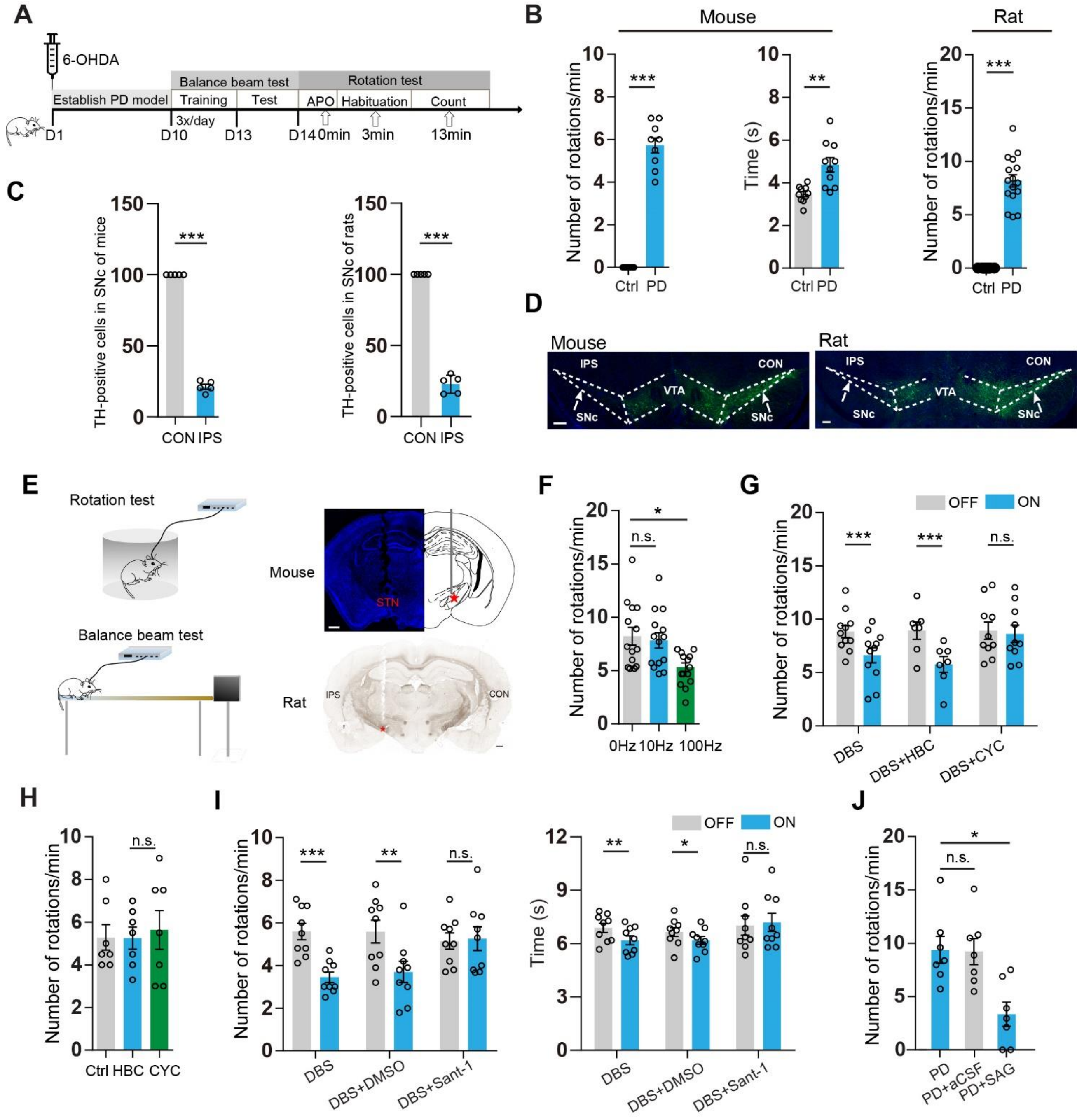

Fig. S1 Shh signaling modulates the corrective effects of STN-DBS on PD motor deficits. **A** Schedule for establishing the PD model and conducting behavioral assays to assess motor deficits. D, Day. **B** Number of APO-induced rotations (left: *n* = 9 mice per group, Mann–Whitney U-test) and the time for passing the beam (middle, *n* = 10 mice per group, Welch's *t*-test) in control (Ctrl) and PD mice. The number of APO-induced rotations in Ctrl and PD rats (right, *n* = 16 rats per group, Mann–Whitney U-test). **C** The relative expression (ipsilateral /contralateral) of TH-positive cells in the SNc of mice

(left) and rats (right). **D** Representative images of anti-TH immunofluorescent staining in the SNc of the mouse (left) and rat (right) after injection of 6-OHDA. Scale bars, 200 μm. **E** Schematics of motor assessment during STN-DBS (left) and representative images of the electrode track in the STN of PD mice (upper, right) and rat (lower, right). Scale bars, 500 μm. **F** Number of rotations (left, n = 14 rats per group, one-way ANOVA with Dunnett's *post-hoc* test) after STN-DBS at the indicated frequencies in PD rats. **G** STN-DBS corrective effects on the number of rotations (n = 7−11 rats per group, two-way ANOVA with Bonferroni's *post-hoc* test) after i.p. injection of Cyc or HBC in the PD rats. **H** Number of rotations after injecting Cyc or HBC in PD mice (n = 7 mice per group, one-way ANOVA with Dunnett's *post-hoc* test). **I** STN-DBS effects on the number of APO-induced rotations (left, n = 9 mice per group, two-way ANOVA with Bonferroni's *post-hoc* test) and the time for passing the beam (right, n = 9 mice per group, two-way ANOVA with Bonferroni's *post-hoc* test) after i.c.v. injection of Sant-1 (0.05 mg/kg) or its solvent (DMSO) in PD mice. **J** Number of rotations (n = 7 rats per group, Kruskal Wallis test with Dunn's *post-hoc* test) after i.c.v. injection of SAG (15 μmol/L in 3 μL) or aCSF in PD rats. For all behavioral experiments, all values are reported as the mean ± SEM from at least three independent experiments. Each circle represents a mouse in **B** (left and middle panels), **C** (left panel), **H**, and **I**; each circle represents a rat in **B** (right panel), **C** (right panel), **F**, **G**, and **J**. *P <0.05, **P <0.01, ***P <0.001, and n.s., no significant difference.

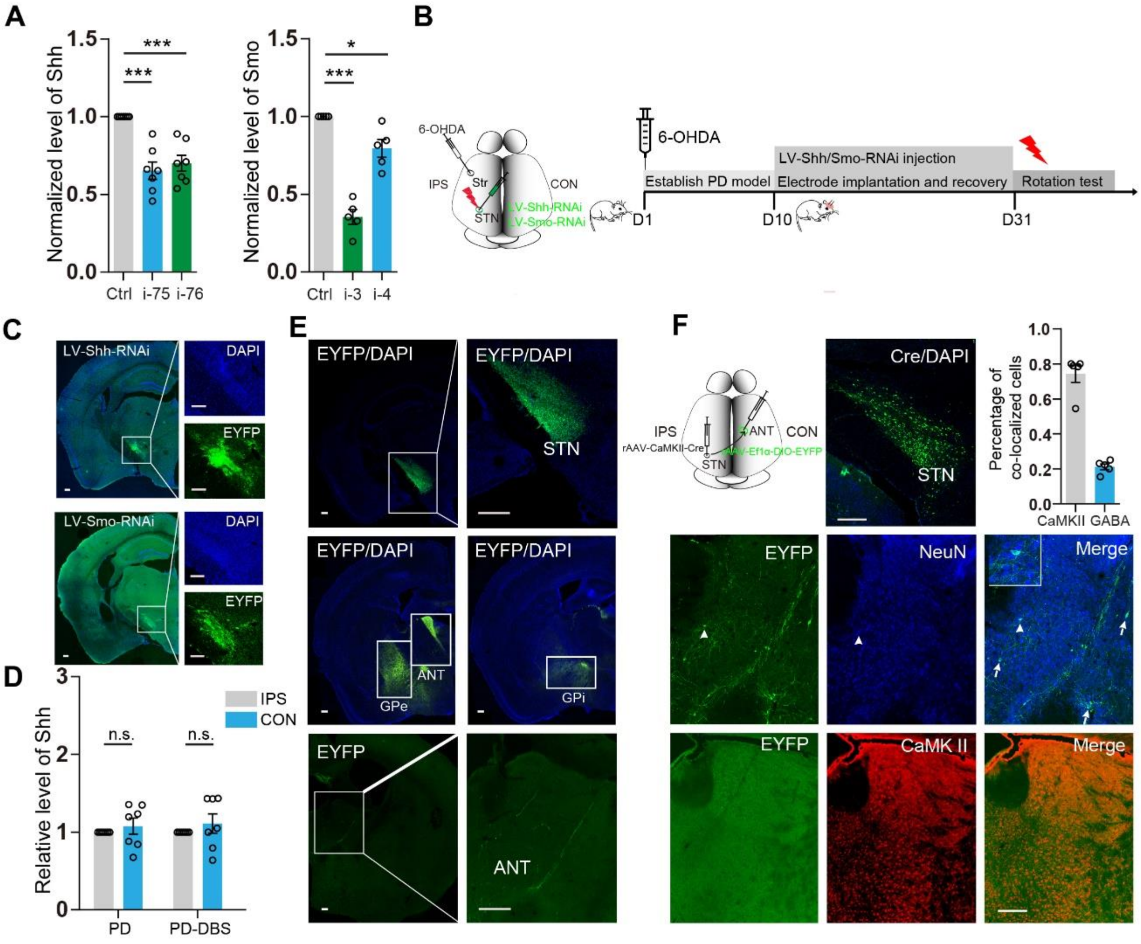

Fig. S2 Contralateral ANT releases Shh upon high-frequency STN-DBS. **A** Analysis of Shh (left, *n* = 7 cultures, one-way ANOVA with Dunnett's *post-hoc* test) and Smo (right, *n* = 5 cultures, one-way ANOVA with Dunnett's *post-hoc* test) in cultured neurons treated with lentivirus (LV)-Shh-RNAi or LV-Smo-RNAi, respectively. Protein expression is normalized to α-tubulin, and protein expression with RNAi is normalized to the control virus. **B** Left: schematic of the electrode implantation and virus injection in the STN of PD mice; right: schedule of STN-DBS effects on the rotations after knocking down Shh or Smo in the STN. **C** Representative images showing EYFP expression in the STN. Scale bars, 200 μm. **D** Levels of Shh released from ipsilateral and contralateral GPi in hemi-parkinsonian mice with or without STN-DBS (*n* = 7 mice, Wilcoxon matched-pairs signed rank test). Shh levels in the contralateral nuclei are normalized to the ipsilateral nuclei in each mouse. **E** Representative images of EYFP expression in the STN and its downstream nuclei after AAV-CaMKII-EYFP injection into the unilateral STN. Scale bars, 500 μm. *n* = 5 mice. **F** Upper left, schematic of dual virus injections. Upper middle, representative image of the STN injected with trans-monosynaptic AAV expressing

Cre recombinase. Upper right Statistics of the percentage of EYFP-positive cells co-localized with CaMKII or GABA. Middle, representative images of EYFP-positive ANT cells stained with anti-NeuN; lower, control images of the virus expressing DIO-EYFP injected into the ANT. Inset: magnified views of arrowhead regions. Scale bars, 200 μm. *n* = 5 mice. For all experiments, all values are reported as the mean ± SEM. Each circle represents a mouse in **C** and **E**; the circle represents neurons in **A**. **P* <0.05, ***P* <0.01, ****P* <0.001, and n.s. no significant difference.

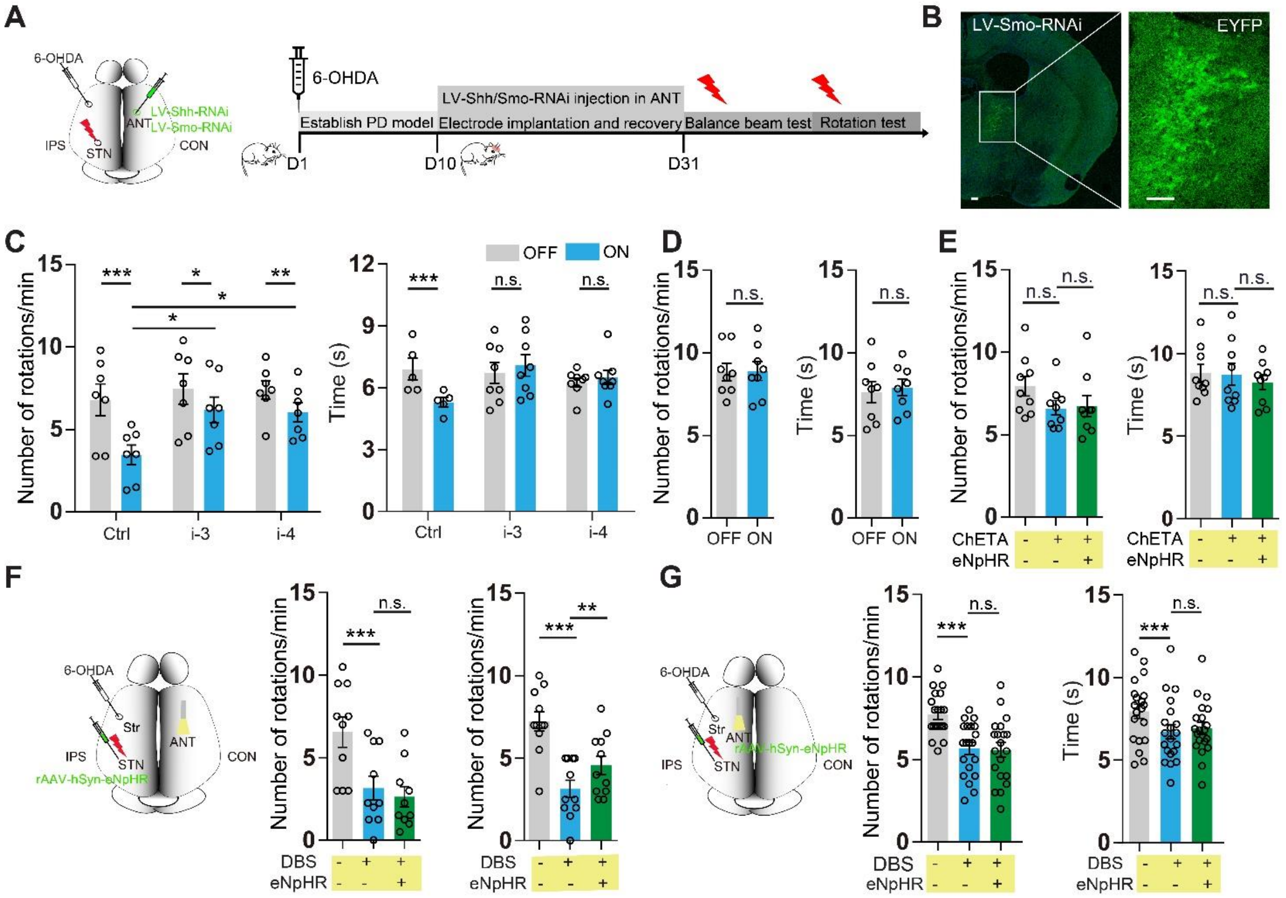

Fig. S3 Shh signaling in the contralateral ANT modulates STN-DBS actions on PD motor deficits. **A** Schedule of testing STN-DBS effects on the motor deficits in PD mice after knocking down Shh or Smo in the ANT. **B** Representative images of LV-Smo-RNAi expression in the contralateral ANT. Scale bars, 200 μm. **C** STN-DBS effects on rotation (left, *n* = 7 mice per group, two-way ANOVA with Bonferroni's *post-hoc* test) and the time for passing the beam (right, *n* = 5–8 mice per group, two-way ANOVA with Bonferroni's *post-hoc* test) after knocking down Smo in the contralateral ANT. **D** Number of rotations (middle, *n* = 8 mice per group, paired two-sided t-test) and the time for passing

the beam (right, n = 8 mice per group, paired two-sided t-test) after low-frequency optical stimulation (470 nm, 10 Hz). **E** Number of rotations (middle, n = 9 mice in each group, one-way ANOVA with Bonferroni's *post-hoc* test) and the time of passing the beam (right, n = 9 mice per group, one-way ANOVA with Bonferroni's *post-hoc* test) after low-frequency optical stimulation (470 nm, 10 Hz) in the ipsilateral STN followed by 590 nm optical stimulation in the contralateral ANT. **F** Left: schematic of DBS, virus injection, and optical fiber implantation into the ipsilateral STN and contralateral ANT of PD mice. Right: STN-DBS effects on rotation after inhibiting the STN-contralateral ANT pathway by expressing eNpHR in the ipsilateral STN and optically stimulating the fibers in the contralateral ANT (n = 10−11 mice per group, one-way ANOVA with Bonferroni's *post-hoc* test). **G** Left: schematic of DBS, virus injection, and optical fiber implantation into the ipsilateral STN and the ipsilateral ANT of PD mice. Right: STN-DBS effects on rotation (n = 20 mice per group, one-way ANOVA with Bonferroni's *post-hoc* test) and the time for passing the beam (n = 20 mice per group, one-way ANOVA with Bonferroni's *post-hoc* test) after inhibiting the STN-ipsilateral ANT pathway by expressing eNpHR in the ipsilateral STN and optically stimulating the fibers in the ipsilateral ANT. For all experiments, all values are reported as the mean ± SEM from at least three independent experiments. Each circle represents a mouse. *P <0.05, **P <0.01, ***P <0.001, and n.s. no significant difference.
